# Supplementary material for: Identification of ephrin-A1–EphA2 signalling as a potential target for fracture prevention
Source: Nat Commun. 2026 Feb 21;17:1988. doi: 10.1038/s41467-026-69863-6 (PMC12932640; doi:10.1038/s41467-026-69863-6)
Supplement: Supplementary file 1 — Supplementary Information [file 41467_2026_69863_MOESM1_ESM.pdf]

Supplementary Figure S1

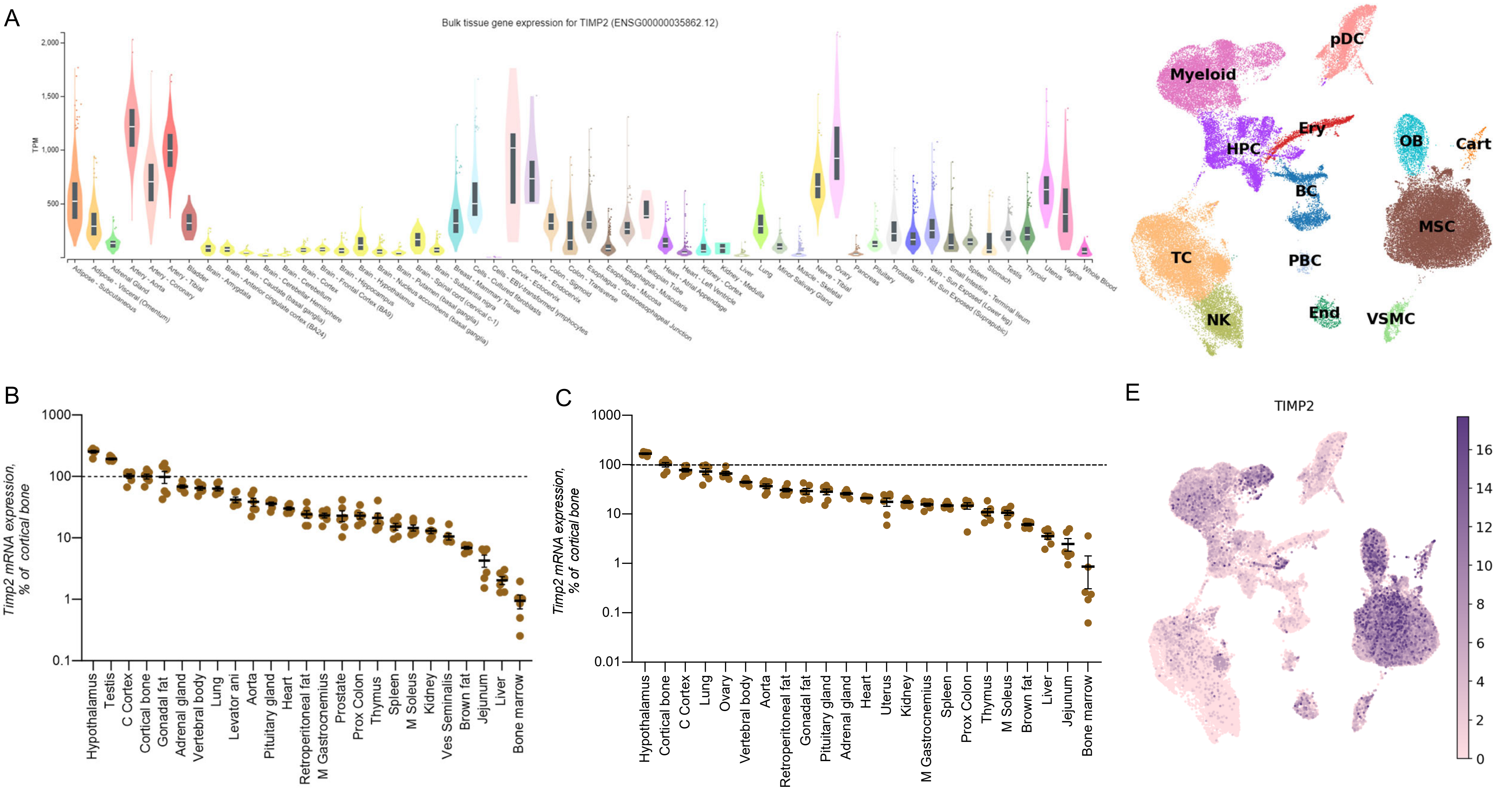

Supplementary Figure S1. Expression patterns of *TIMP2* in humans and mice.

(A) *TIMP2* mRNA expression in 54 human tissues, available at the GTEx portal (https://gtexportal.org/home/). Box plots are shown as median and 25th and 75th percentiles. (B, C) *Timp2* mRNA expression pattern in several tissues from 12-week-old male (B, n = 6) and female (C, n = 6) C57BL/6N wild type mice. Data are presented as % of the expression in cortical bone with individual values in the scatter dot plot. Lines indicate mean ± standard error. (D) Embedding of the combined human bone marrow scRNA-seq atlas. Broad cell identities according to marker gene expression. (E) Single-cell gene expression levels of TIMP2, normalized by sequencing depth. BC, B-cells; Cart, chondrocytes; End, endothelial cells; Ery, erythrocytes; HPC, hematopoietic progenitors; MSC, mesenchymal stem cells; Myeloid, myeloid cells; NK, natural killer cells; OB, osteoblasts; PBC, plasma B-cells; TC, T-cells; VSMC, vascular smooth muscle cells; pDC, plasmacytoid dendritic cells.

Supplementary Figure S2

A

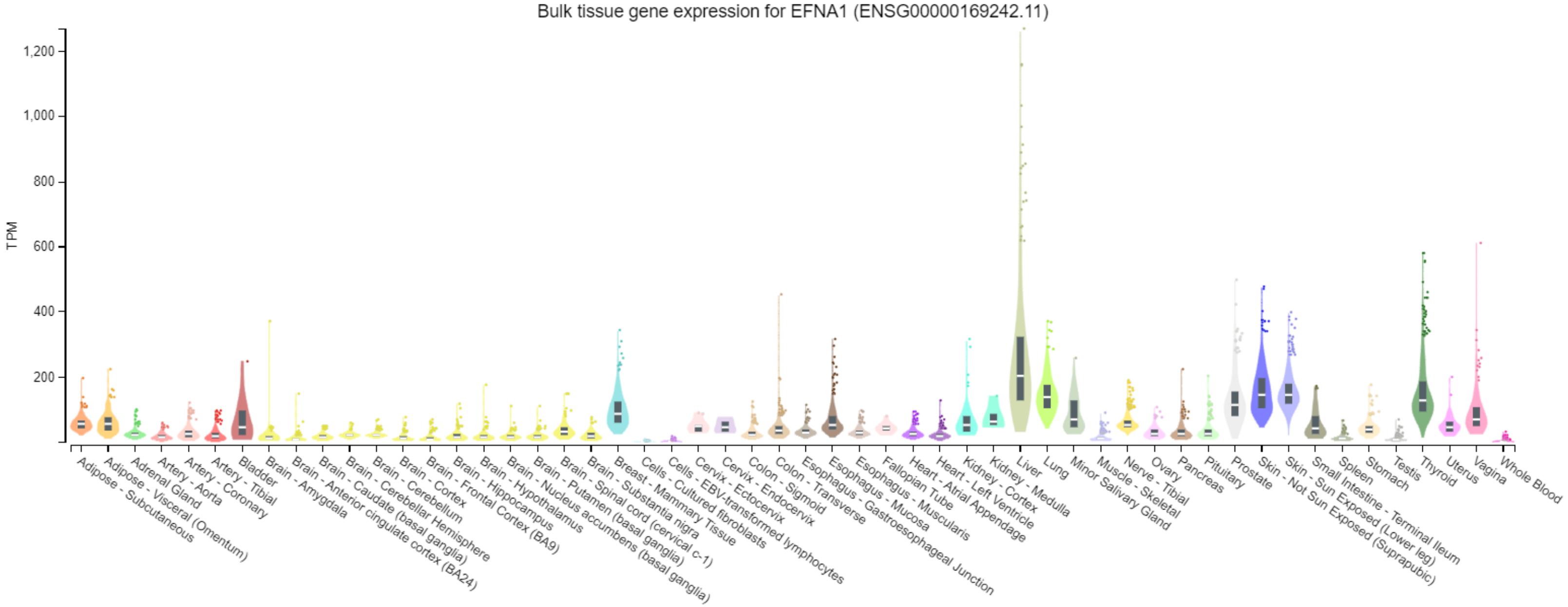

B

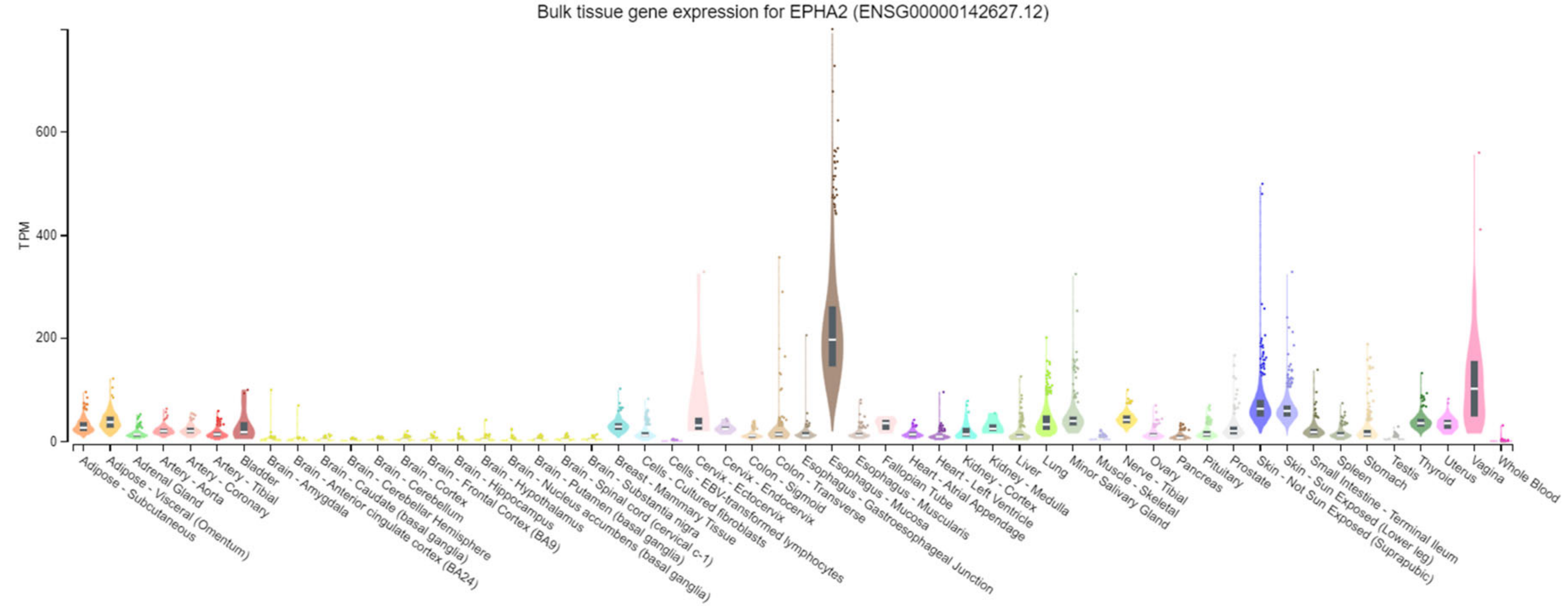

Supplementary Figure S2. *EFNA1* and *EPHA2* mRNA expression in 54 human tissues.

(A) *EFNA1* and (B) *EPHA2* mRNA expression in 54 human tissues, available at the GTEx portal (https://gtexportal.org/home/). Box plots are shown as median and 25th and 75th percentiles.

Supplementary Figure S3

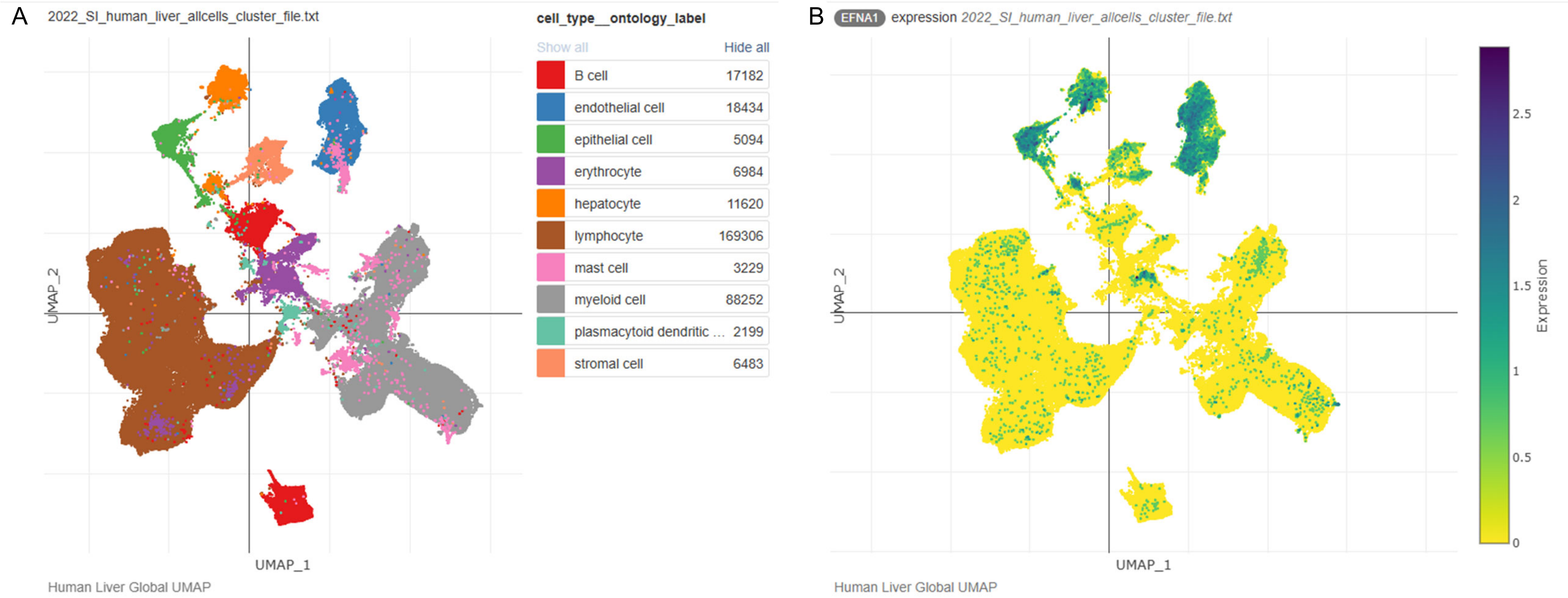

Supplementary Figure S3. Cell cluster plot and *EFNA1* gene expression scatter plot.

(A) Cell cluster plot and (B) *EFNA1* gene expression scatter plot of single-cell RNA sequencing data of human liver cells. Fabre T. et al., *Sci. Immunol.* 8, eadd8945(2023). DOI:10.1126/sciimmunol.add8945 https://singlecell.broadinstitute.org/single_cell/study/SCP2154/

Supplementary Figure S4

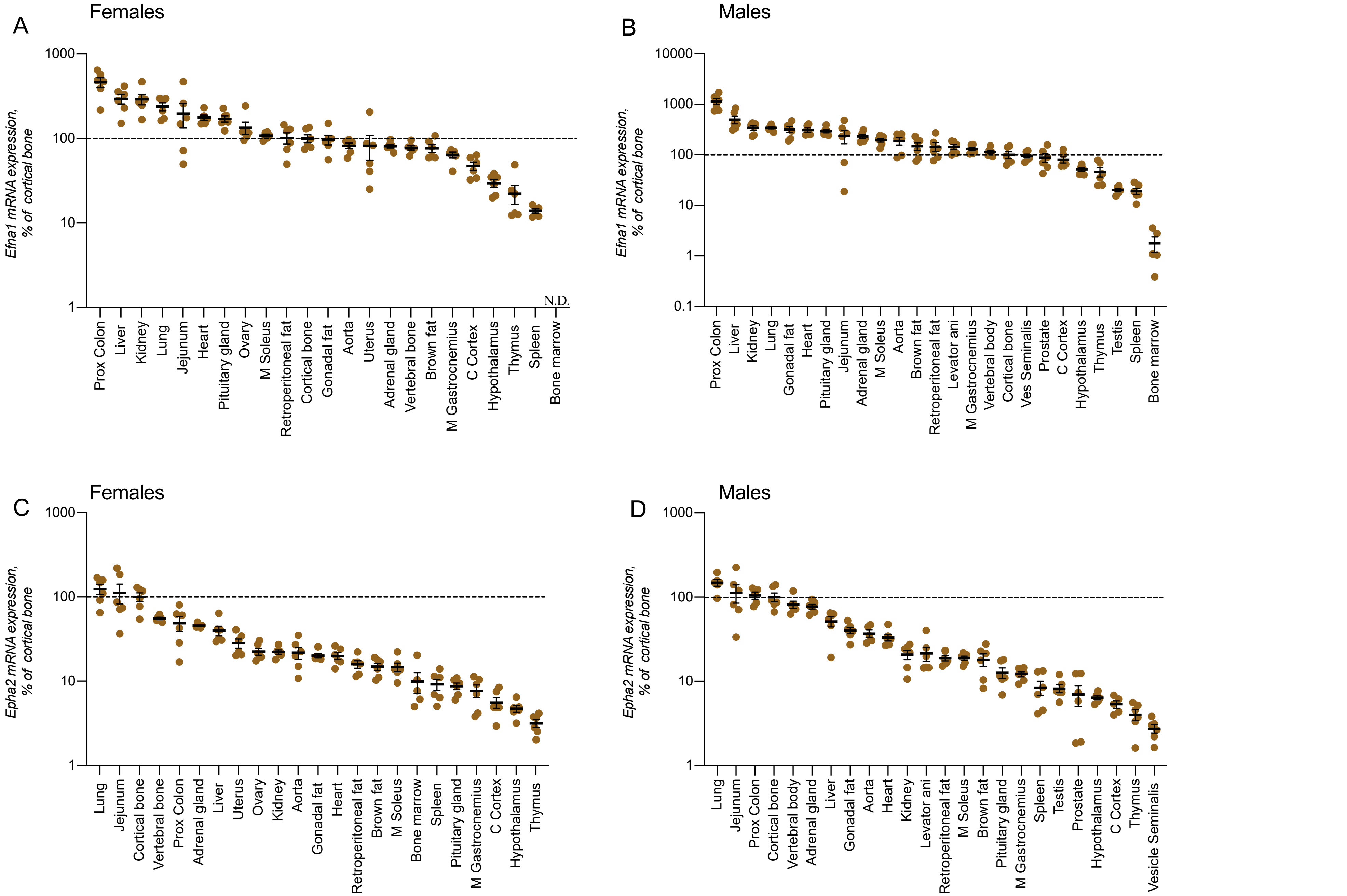

Supplementary Figure S4. *Efna1* and *Epha2* mRNA expression in mouse tissues.

(A, B) *Efna1* mRNA expression in various tissues from 12-week-old female (A) and male (B) C57BL/6N wild type mice (n = 6). (C, D) *Epha2* mRNA expression in various tissues from12-week-old female (C) and male (D) C57BL/6N wild type mice (n = 6). Data are presented as % of the expression in cortical bone with individual values in the scatter dot plot. Lines indicate mean ± standard error.

Supplementary Figure S5

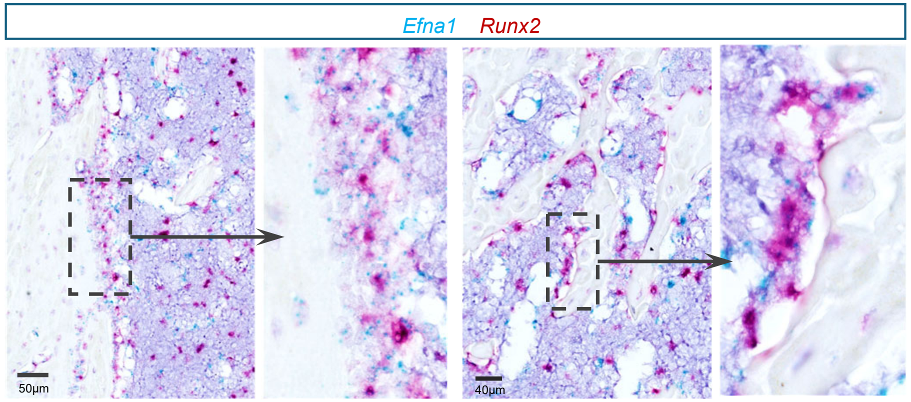

Supplementary Figure S5. Colocalization of *Efna1* and *Runx2* mRNA in adult mouse long bone revealed by chromogenic *in situ* hybridization (CISH).

Chromogenic *in situ* hybridization was performed to simultaneously detect *Efna1* and *Runx2* mRNA expression in mouse long bone sections. *Efna1* mRNA was visualized using a green chromogen, and *Runx2* mRNA, a marker of osteoprogenitor cells, was detected using a red chromogen. A high degree of signal overlap was observed between the two transcripts, showing that *Efna1* is expressed in *Runx2*-positive osteoprogenitor cells. Experiments were performed on bone sections from six independent adult mice and repeated in a separate session, with consistent observations across all replicates.

Supplementary Figure S6

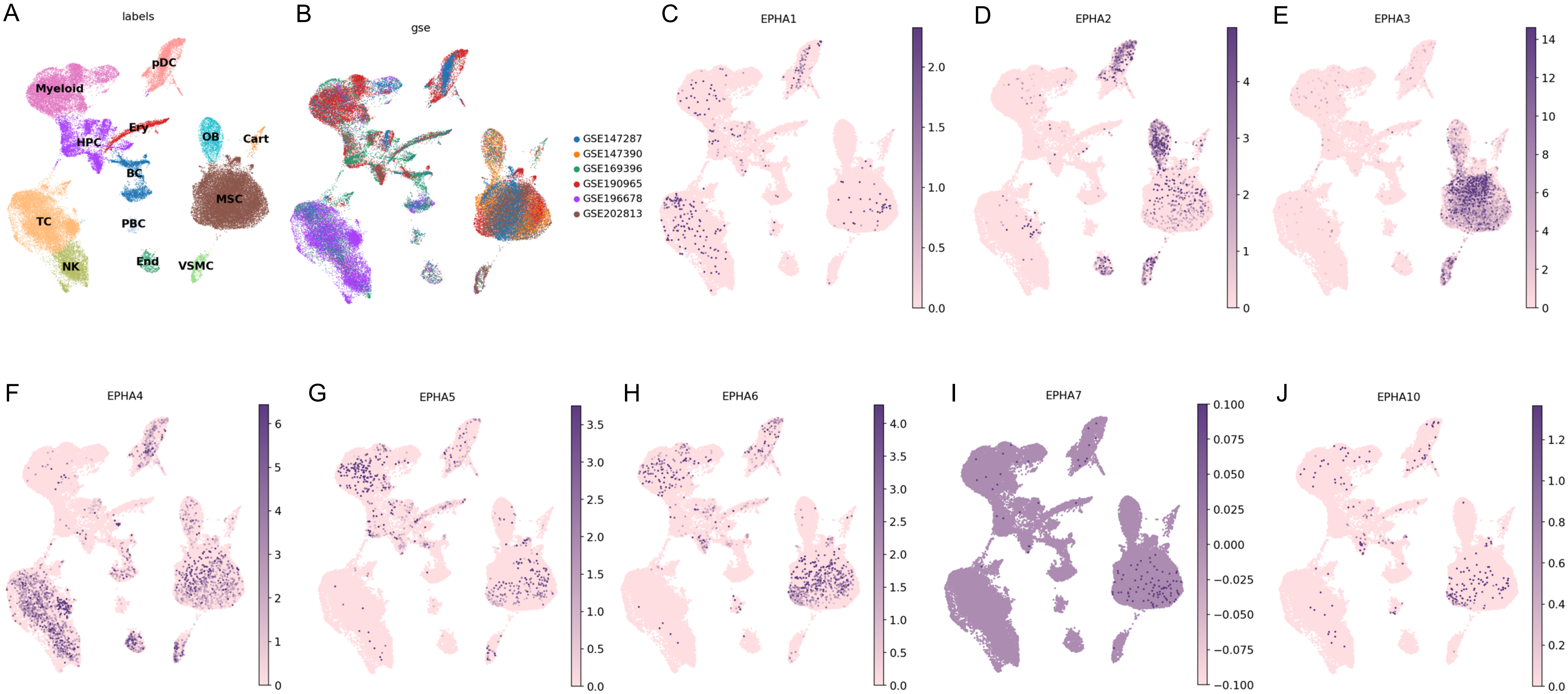

Supplementary Figure S6. Single-cell mRNA sequencing-based analysis of human bone marrow in relation to various EPHAs.

To evaluate gene expression in different rare human non-hematopoietic bone marrow populations, a special atlas was created by combining several single-cell RNA sequencing experiments enriched for cell types of interest. (A-J) Single-cell RNA sequencing data (scRNA-seq) of expression of different EPHA receptors in human bone marrow. (A) Embedding of the combined human bone marrow scRNA-seq atlas. Broad cell identities according to marker gene expression. (B) Embedding displaying combined contribution by different data sources. (C-J) Feature plots showing gene expression levels of different EPHA receptors, normalized by sequencing depth.

BC = B-cells; Cart = chondrocytes, End = endothelial cells, Ery = erythrocytes, HPC = hematopoietic progenitors, MSC = mesenchymal stem cells, Myeloid = myeloid cells, NK = natural killer cells, OB= osteoblasts, PBC = plasma B-cells, TC = T-cells, VSMC = vascular smooth muscle cells, pDC = plasmacytoid dendritic cells.

Supplementary Figure S7

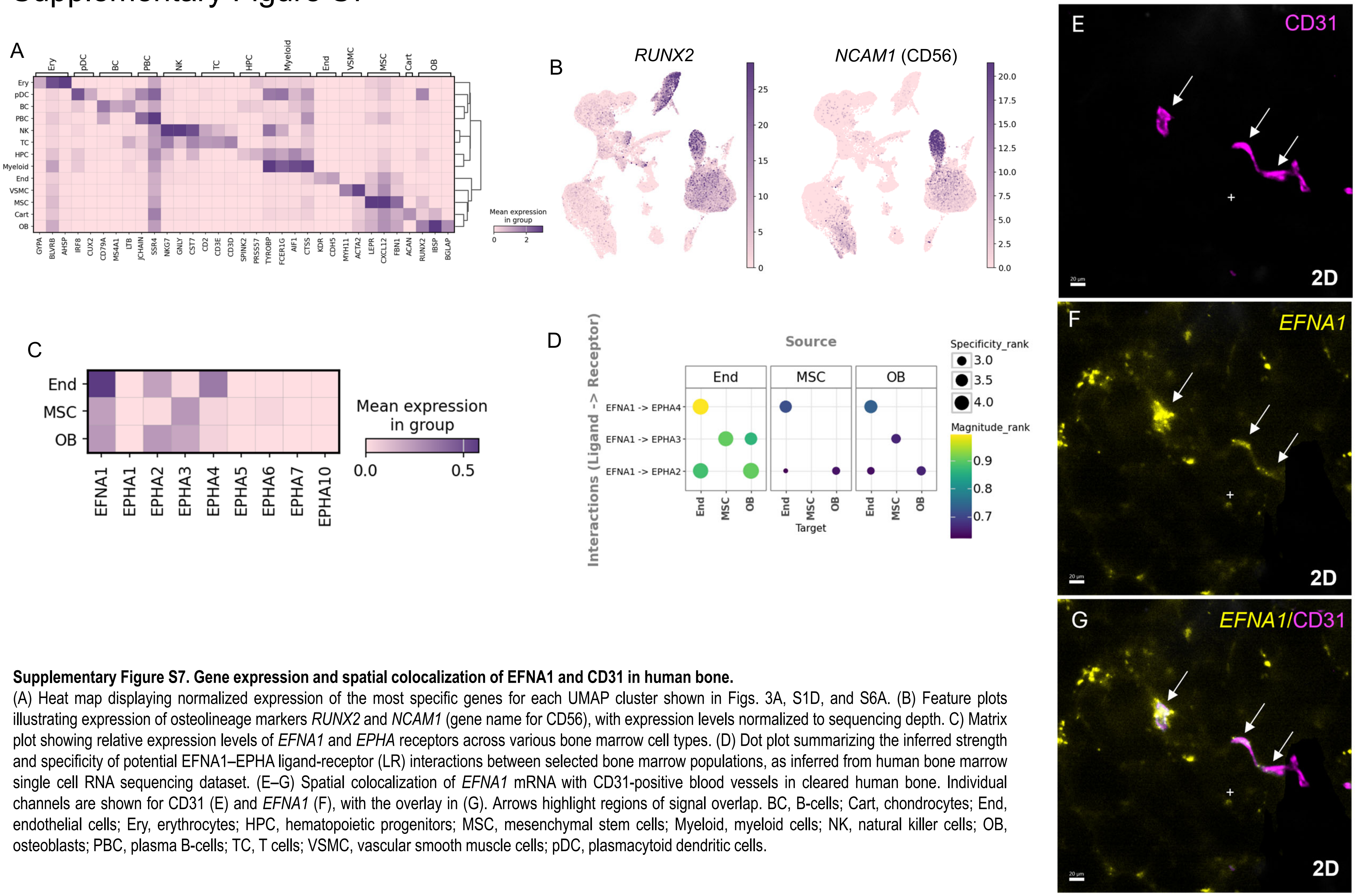

Supplementary Figure S7. Gene expression and spatial colocalization of EFNA1 and CD31 in human bone.
(A) Heat map displaying normalized expression of the most specific genes for each UMAP cluster shown in Figs. 3A, S1D, and S6A. (B) Feature plots illustrating expression of osteolineage markers *RUNX2* and *NCAM1* (gene name for CD56), with expression levels normalized to sequencing depth. C) Matrix plot showing relative expression levels of *EFNA1* and *EPHA* receptors across various bone marrow cell types. (D) Dot plot summarizing the inferred strength and specificity of potential EFNA1–EPHA ligand-receptor (LR) interactions between selected bone marrow populations, as inferred from human bone marrow single cell RNA sequencing dataset. (E–G) Spatial colocalization of *EFNA1* mRNA with CD31-positive blood vessels in cleared human bone. Individual channels are shown for CD31 (E) and *EFNA1* (F), with the overlay in (G). Arrows highlight regions of signal overlap. BC, B-cells; Cart, chondrocytes; End, endothelial cells; Ery, erythrocytes; HPC, hematopoietic progenitors; MSC, mesenchymal stem cells; Myeloid, myeloid cells; NK, natural killer cells; OB, osteoblasts; PBC, plasma B-cells; TC, T cells; VSMC, vascular smooth muscle cells; pDC, plasmacytoid dendritic cells.

Supplementary Figure S8

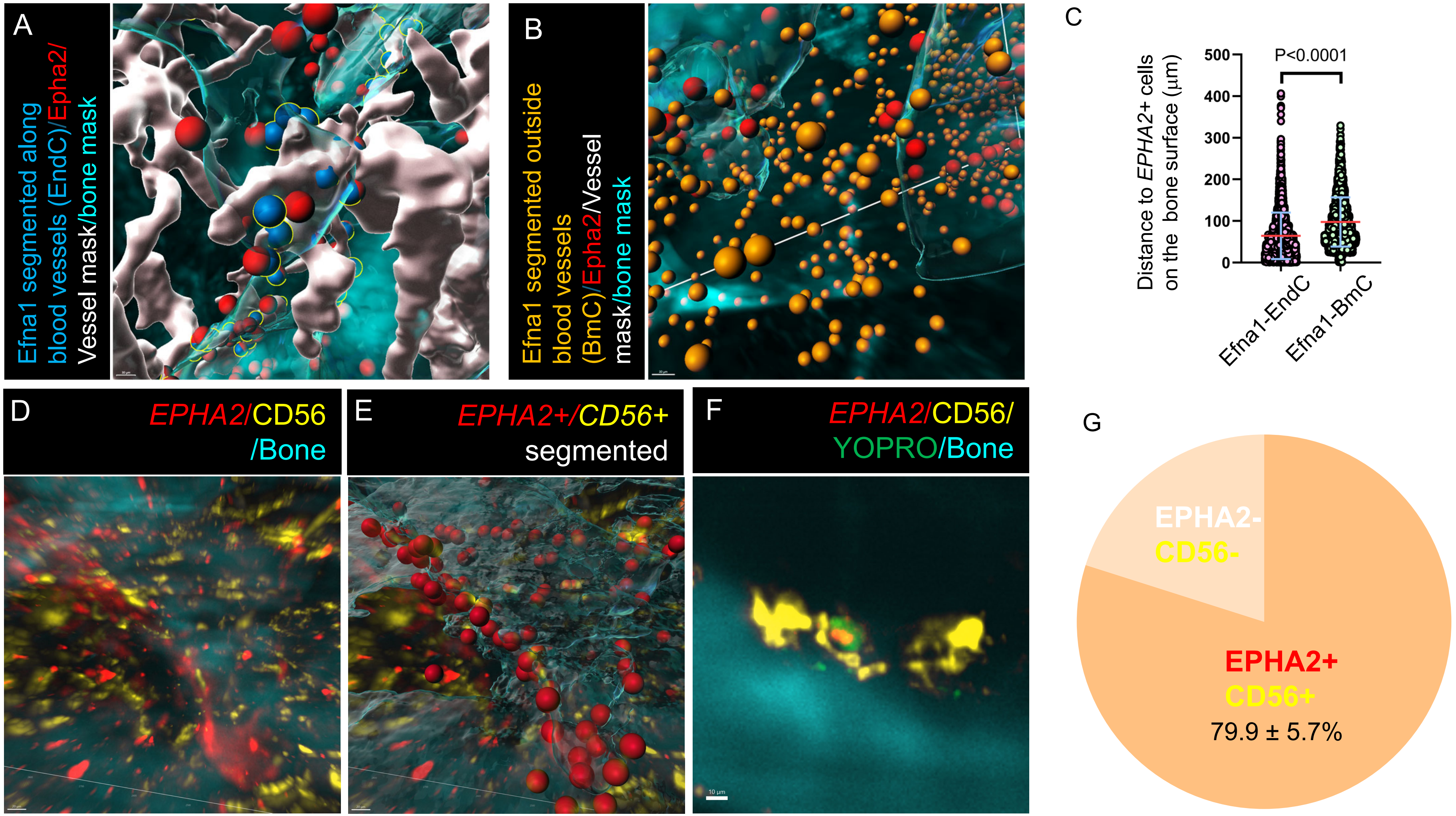

Supplementary Figure S8. Integrated spatial analysis of EFNA1- and EPHA2-expressing cells in cleared human bone.
(A, B) Segmented 3D scans of cleared human bone samples stained for *EFNA1* mRNA, *EPHA2* mRNA, CD31 (blood vessels), and bone matrix (visualized by autofluorescence). EFNA1-positive cells located within 20 µm of blood vessels (Efna1-EndC) are shown as blue spheres (A), while EFNA1-positive cells located beyond 20 µm from both blood vessels and the bone surface (Efna1-BmC) are shown as orange spheres (B). EPHA2-positive cells located within 20 µm of the bone surface are shown as red spheres in both panels. Bone and vessel masks are pseudocolored cyan and grey, respectively. (C) Quantification of the distance between Efna1-EndC and Efna1-BmC cells and EPHA2-positive cells on the bone surface. Statistical analysis was performed using a two-sided Student's t test. (D) *EPHA2* mRNA (pseudocolored red) is shown together with CD56 protein (pseudocolored yellow) and bone matrix (pseudocolored cyan). A maximum intensity projection of the 3D scan is shown. (E) EPHA2- and CD56- double-positive cells are represented as red spheres, with sphere size indicating their distance to the frontal plane. Maximum intensity projection of the segmented 3D scan is shown. (F) Optical section corresponding to panel D. YOPRO-stained nuclei (pseudocolored green) were omitted from panel D for visual clarity. (G) Quantification of EPHA2- and CD56-positive cells located within 20 µm of the bone surface. Data are obtained from 3 femoral heads (3 patients) and represent mean ± SE (panels G) and integration of 3 patients (panel C).
