# Supplementary material for: Identification of ephrin-A1–EphA2 signalling as a potential target for fracture prevention
Source: Nat Commun. 2026 Feb 21;17:1988. doi: 10.1038/s41467-026-69863-6 (PMC12932640; doi:10.1038/s41467-026-69863-6)
Supplement: Supplementary file 2 — Description of Additional Supplementary Files [file 41467_2026_69863_MOESM2_ESM.pdf]

Description of Additional Supplementary Files

Supplementary Data 1 Identification of causal circulating proteins for forearm fractures using a Mendelian randomization (MR) pipeline

Supplementary Data 2 Colocalization analyses between nine selected proteins and forearm fractures

Supplementary Data 3 Separate and combined associations for the FAM3C signal (rs138090420) and a known forearm fracture-related WNT16 signal (rs2908007) with forearm fractures in UK Biobank

Supplementary Data 4 The causal associations for three novel fracture related circulating proteins with estimated BMD (eBMD) in the heel by ultrasound and total body BMD by DXA as determined by Mendelian randomization (MR)

Supplementary Data 5 Mendelian randomization (MR) sensitivity analyses of the association between genetically determined circulating EFNA1 and fracture risk using alternative genetic instruments and outcomes

Supplementary Data 6 The probe sequences used for the detection of EFNA1 and EPHA2 in 3D spatial transcriptomics
