# Supplementary material for: Identification of ephrin-A1–EphA2 signalling as a potential target for fracture prevention
Source: Nat Commun. 2026 Feb 21;17:1988. doi: 10.1038/s41467-026-69863-6 (PMC12932640; doi:10.1038/s41467-026-69863-6)
Supplement: Supplementary file 4 — Reporting Summary [file 41467_2026_69863_MOESM4_ESM.pdf]

nature portfolio

Corresponding author(s): Claes Ohlsson and Sofia Movérare-Skrtic

Last updated by author(s): Dec 11, 2025

Reporting Summary

Nature Portfolio wishes to improve the reproducibility of the work that we publish. This form provides structure for consistency and transparency in reporting. For further information on Nature Portfolio policies, see our Editorial Policies and the Editorial Policy Checklist.

Statistics

For all statistical analyses, confirm that the following items are present in the figure legend, table legend, main text, or Methods section.

n/a	Confirmed	
☐	☒	The exact sample size (*n*) for each experimental group/condition, given as a discrete number and unit of measurement
☐	☒	A statement on whether measurements were taken from distinct samples or whether the same sample was measured repeatedly
☐	☒	The statistical test(s) used AND whether they are one- or two-sided<br>*Only common tests should be described solely by name; describe more complex techniques in the Methods section.*
☒	☐	A description of all covariates tested
☐	☒	A description of any assumptions or corrections, such as tests of normality and adjustment for multiple comparisons
☐	☒	A full description of the statistical parameters including central tendency (e.g. means) or other basic estimates (e.g. regression coefficient) AND variation (e.g. standard deviation) or associated estimates of uncertainty (e.g. confidence intervals)
☐	☒	For null hypothesis testing, the test statistic (e.g. *F*, *t*, *r*) with confidence intervals, effect sizes, degrees of freedom and *P* value noted<br>*Give P values as exact values whenever suitable.*
☒	☐	For Bayesian analysis, information on the choice of priors and Markov chain Monte Carlo settings
☒	☐	For hierarchical and complex designs, identification of the appropriate level for tests and full reporting of outcomes
☒	☐	Estimates of effect sizes (e.g. Cohen's *d*, Pearson's *r*), indicating how they were calculated

Our web collection on statistics for biologists contains articles on many of the points above.

Software and code

Policy information about availability of computer code

Data collection	QuantStudio 3
Data analysis	GraphPad Prism v10.4.0<br>Imaris v10.1.0 or 10.2<br>ImarisConverter v10.0<br>Python v3.9.16. Packages: (python 3.9, anndata 0.9.0, liana 1.1.0, matplotlib 3.8.4, numpy 1.26.4, pandas 2.3.3, scanpy 1.10.1, pymultimap 0.0.8, scvi 0.6.8)<br>R v4.4.3 (https://cran.r-project.org/). Packages: MendelianRandomization, LDlinkR, dplyr.<br>pwcoco https://github.com/jwr-git/pwcoco

For manuscripts utilizing custom algorithms or software that are central to the research but not yet described in published literature, software must be made available to editors and reviewers. We strongly encourage code deposition in a community repository (e.g. GitHub). See the Nature Portfolio guidelines for submitting code & software for further information.

Data

Policy information about availability of data

All manuscripts must include a data availability statement. This statement should provide the following information, where applicable:

- Accession codes, unique identifiers, or web links for publicly available datasets
- A description of any restrictions on data availability
- For clinical datasets or third party data, please ensure that the statement adheres to our policy

All GWAS summary statistics for the exposures and outcomes in the Mendelian randomization analyses are available online: circulating proteins https://www.decode.com/summarydata/, eBMD and total body BMD http://www.gefos.org/, forearm fractures at the GWAS Catalog under study accession number GCST90281273 (https://www.ebi.ac.uk/gwas). The human bone marrow single-cell RNA sequencing atlas can be explored at the CellxGene Portal: https://cellxgene.cziscience.com/collections/0391c84c-d57d-4741-9277-e4d58f9a3d0c
and primary data used in the present study are available at https://www.ncbi.nlm.nih.gov/geo/query/acc.cgi?acc=GSE147287; https://www.ncbi.nlm.nih.gov/geo/query/acc.cgi?acc=GSE147390; https://www.ncbi.nlm.nih.gov/geo/query/acc.cgi?acc=GSE169396; https://www.ncbi.nlm.nih.gov/geo/query/acc.cgi?acc=GSE190965; https://www.ncbi.nlm.nih.gov/geo/query/acc.cgi?acc=GSE196678; https://www.ncbi.nlm.nih.gov/geo/query/acc.cgi?acc=GSE202813.
Source data are provided in this paper.

Research involving human participants, their data, or biological material

Policy information about studies with human participants or human data. See also policy information about sex, gender (identity/presentation), and sexual orientation and race, ethnicity and racism.

Reporting on sex and gender	Since the risk of fracture differ between males and females, analyses of UK Biobank have been adjusted for self-reported gender at baseline examination.
Reporting on race, ethnicity, or other socially relevant groupings	All analyses including UK Biobank participants have considered self-reported ethnicity.
Population characteristics	For exposure in Mendelian randomization, we have used summary statistics from Eldjarn, G.H. et al. Large-scale plasma proteomics comparisons through genetics and disease associations. Nature 622, 348-358 (2023). This study is based upon UK Biobank. The UK Biobank is a large prospective cohort study of approximately a half-million adult (ages 40–69 years) participants living in the United Kingdom with genotype, phenotype, and linked health record data, recruited from 22 centers across the United Kingdom in 2006–2010. Please see further details in that article.<br><br>For outcome in Mendelian randomization (forearm fracture), we have used summary statistics from Nethander, M. et al. An atlas of genetic determinants of forearm fracture. Nat Genet 55, 1820-1830 (2023). Please see further details in that article.<br><br>For outcome in Mendelian randomization (fracture at any skeletal site), we have used summary statistics from Morris, J.A. et al. An atlas of genetic influences on osteoporosis in humans and mice. Nat Genet 51, 258-266 (2019). Please see further details in that article.
Recruitment	UK Biobank participants were recruited between 2006 and 2010.
Ethics oversight	For the human Mendelian randomization analyses, we used publicly available GWAS summary statistics. These GWASs have previously been published with relevant ethical approvals. The histology work with human bone samples was approved by the Swedish Ethics Review Authority (Etikprövningsmyndigheten Dnr 2022-01977-02).

Note that full information on the approval of the study protocol must also be provided in the manuscript.

Field-specific reporting

Please select the one below that is the best fit for your research. If you are not sure, read the appropriate sections before making your selection.

☒ Life sciences ☐ Behavioural & social sciences ☐ Ecological, evolutionary & environmental sciences

For a reference copy of the document with all sections, see nature.com/documents/nr-reporting-summary-flat.pdf

Life sciences study design

All studies must disclose on these points even when the disclosure is negative.

Sample size	Sample size for the Mendelian randomization analyses were chosen on the basis of all data available at the time for analysis. No statistical method was used to predetermine sample size. We designed our experiments based on previous experience. All samples sizes are given in each figure legend or methods.

Data exclusions	No data were excluded from the analyses unless technical issues.
Replication	All samples sizes are given in each figure legend or methods.
Randomization	Not applicable (it is a population-based longitudinal cohort study and not a randomized clinical study).
Blinding	Not applicable (it is a population-based longitudinal cohort study and not a randomized clinical study).

Reporting for specific materials, systems and methods

We require information from authors about some types of materials, experimental systems and methods used in many studies. Here, indicate whether each material, system or method listed is relevant to your study. If you are not sure if a list item applies to your research, read the appropriate section before selecting a response.

Materials & experimental systems

n/a	Involved in the study
☐	☒ Antibodies
☒	☐ Eukaryotic cell lines
☒	☐ Palaeontology and archaeology
☐	☒ Animals and other organisms
☒	☐ Clinical data
☒	☐ Dual use research of concern
☒	☐ Plants

Methods

n/a	Involved in the study
☒	☐ ChIP-seq
☒	☐ Flow cytometry
☒	☐ MRI-based neuroimaging

Antibodies

Antibodies used	anti-CD31 (AF3628, R&D systems, 1:100)<br>anti-RUNX2 (ab192256, Abcam, 1:100)<br>anti-CD56 (AF2408, R&D systems; 1:100)
Validation	All antibodies were validated by the supplier.

Animals and other research organisms

Policy information about studies involving animals; ARRIVE guidelines recommended for reporting animal research, and Sex and Gender in Research

Laboratory animals	Total body bone minearl density had been evaluated in Efna1-/- mice (Efna1tm1a(EUCOMM)Wtsi allele) and Epha2-/- mice(Epha2em1(IMPC)Mbp allele) produced by the International Mouse Phenotyping Consortium (IMPC) (www.mousephenotype.org) at 14 weeks of age. Gene expression in different tissues was analysed in 12-week-old female and male C57BL/6N wildtype mice. To investigate the regulation of Efna1 and EphA2 expression in bone tissue under pathological and anabolic conditions, a range of mouse models was employed. A systemic inflammation-induced bone loss model was studied in 9-week-old female C57BL/6N mice. The age-induced bone loss model involved 15-month-old and 2-month-old female C57BL/6JRj mice from the same breeder (Janvier Labs, France). The vitamin A-induced bone loss model was conducted in 9-week-old female C57BL/6N mice, while the postmenopausal osteoporosis model used 13-week-old female mice that underwent either ovariectomy or sham surgery. Bone anabolic responses were assessed using a loading-induced bone formation model in 13-week-old female mice, and a PTH-induced bone formation model in 11-week-old male mice.
Wild animals	The study did not involve wild animals.
Reporting on sex	Sex was not specifically considered in the study design or analysis; however, all data are reported disaggregated by sex. Total body bone mineral density had been evaluated in female and male EphA2+/+ and EphA2-/- mice and in female and male Efna1+/+ and Efna1-/- mice. Gene expression in different tissues was analysed in female and male mice. Efna1 and EphA2 expression in bone tissue was assessed in female mice following systemic inflammation-induced, age-induced, vitamin A-induced, and ovariectomy-induced bone loss, as well as in a loading-induced bone formation model. Male mice were used for the PTH-induced bone formation model.
Field-collected samples	The study did not involve samples collected from the field.
Ethics oversight	All animal procedures performed at the University of Gothenburg were approved by the Gothenburg Animal Research Ethics Committee, Sweden.

Note that full information on the approval of the study protocol must also be provided in the manuscript.

Plants

Seed stocks	*Report on the source of all seed stocks or other plant material used. If applicable, state the seed stock centre and catalogue number. If plant specimens were collected from the field, describe the collection location, date and sampling procedures.*
Novel plant genotypes	*Describe the methods by which all novel plant genotypes were produced. This includes those generated by transgenic approaches, gene editing, chemical/radiation-based mutagenesis and hybridization. For transgenic lines, describe the transformation method, the number of independent lines analyzed and the generation upon which experiments were performed. For gene-edited lines, describe the editor used, the endogenous sequence targeted for editing, the targeting guide RNA sequence (if applicable) and how the editor was applied.*
Authentication	*Describe any authentication procedures for each seed stock used or novel genotype generated. Describe any experiments used to assess the effect of a mutation and, where applicable, how potential secondary effects (e.g. second site T-DNA insertions, mosiacism, off-target gene editing) were examined.*
